# ‘I can feel sad about it and I can worry, but inside I know everything happens for a reason’: personal experiences in the aftermath of the March 15 Christchurch mosque attacks

**DOI:** 10.1192/bjo.2024.791

**Published:** 2024-10-11

**Authors:** Shaystah Dean, Kate Eggleston, Fareeha Ali, Zimna Thaufeeg, Hayley Wells, Julie Zarifeh, Ruqayyah Sulaiman-Hill, Caroline Bell, Marie Crowe

**Affiliations:** Department of Psychological Medicine, University of Otago Christchurch, New Zealand

**Keywords:** Terror attacks, pscyhosocial impacts, faith, post-traumatic growth, qualitative studies

## Abstract

**Background:**

On 15 March 2019, a white supremacist gunman sequentially attacked two mosques in Christchurch, New Zealand, killing 51 people aged from 3 to 77 years and bullet-injuring 40 more. Approximately 250 people survived the atrocity, and many more family and community members have been directly or indirectly affected.

**Aim:**

To develop an understanding of the personal experiences of some of those affected, including effects on daily life and well-being, in the 18–30 months following the attacks.

**Method:**

Qualitative thematic analysis of semi-structured interviews with 21 men and women from September 2020 to August 2021 was performed. Participants were drawn from a larger quantitative study and included injured, bereaved, witnesses, family members and those from the wider Muslim community in Christchurch.

**Results:**

Four superordinate themes were identified: being overwhelmed in the midst of chaos; experiencing silent and enduring impact; living similarly, but differently; and gaining meaning and growth. These themes captured ongoing distress inclusive of physical symptoms, family and community relationship dynamics and connectedness, secondary stressors, and diversity in coping and growth. For most, the centrality of Islam as a faith tradition was woven throughout.

**Conclusion:**

Consistent with previous literature, post-trauma reactions were pervasive and varied. This appeared to be compounded by secondary stressors in this cohort, such as sociopolitical circumstances, demographic diversity, the COVID-19 pandemic and justice processes. Findings also revealed a strong spiritual thread in the experiences of this minority faith community, shedding light on a complex interaction between recovery and post-traumatic growth.

On 15 March 2019, a white supremacist terrorist attacked two mosques in Christchurch, New Zealand. Within minutes, 51 people were killed and 40 were shot. The attacks were witnessed by at least 250 people and were live-streamed on social media, leading to high levels of exposure within the Christchurch Muslim community and beyond. The scale and violence of this act of terrorism is unprecedented in recent New Zealand history.^1^ The affected community is a diverse community that had religion as a unifying feature.

Studies have shown high prevalence of post-traumatic stress disorder (PTSD) and other mental health conditions^2,3^ after mass shootings. They also report higher rates of adverse mental health outcomes following intentional traumatic events (such as terrorist attacks) compared with non-intentional ones (such as natural disasters).^2^ Many studies have focused on outcomes for individuals directly affected, but family members are also vulnerable to long-lasting impacts including prolonged grief,^4^ reduced work and school functioning,^5^ and increased risk of mental health disorders.^6^

Recent terrorist attacks with religious or racial motivations targeting specific or minority populations have included: attacks at Utoya Island and Oslo, Norway;^7^ Sikh worshippers at prayer in Wisconsin in 2012;^8^ Muslim worshippers attacked during evening prayers in Canada in 2017;^9^ Jewish worshipers at the Tree of Life Synagogue in Pittsburgh in 2018;^10^ and worshippers killed by bomb attacks during Easter church services in Sri Lanka in April 2019.^11^ Although the majority of the extant literature is from quantitative studies, qualitative findings have been reported following terrorist acts in London,^12^ Manchester,^13^ Norway,^14^ Bali,^15^ New York,^16^ Columbine^17^ and Brussels.^18^ These have provided additional and often complementary insights into poorly understood and complex areas. For example, they have described how varied reports of distress are, how these change over time^13^ and survivors’ experience of supports provided.^16^ Although informative, most of this literature relates to studies conducted in Western countries, and there has been relatively little consideration of ethnic or religious factors, despite evidence that these can have important roles in understanding or mitigating psychological sequelae^19^ and that better appreciation and validation of racially minoritised experiences can improve mental healthcare.^20^

The importance of understanding how individuals make sense of their experiences and are able to live full lives after such events has been increasingly recognised as a topic of interest.^13^ The role of supports and the value of feeling socially connected have been consistently associated with positive outcomes after traumatic events.^21^ The importance of religion in facilitating post-traumatic growth has also recently been examined in qualitative studies in both Muslim-majority^22^ and Western contexts.^23^ These identified that survivors who were theistic believers engaged in post-disaster religious meaning-making in which they turned to God and theodicies to appraise the disaster's cause and purpose and the effects on people's religious attachments.^23^ A growing body of literature points to a relationship between religion and disaster outcomes that is context dependent and influenced by individual and community-level variables.^24^ Most studies suggest that religion serves as a positive resource for disaster survivors, although more research is needed to elucidate findings that show both positive and negative mental health outcomes associated with religious appraisals and spiritual experiences.^25^ Efforts to engage in religious practices and meaning-making are commonplace in the aftermath of difficult life events in both Western and non-Western settings.^26^

Although there is evidence that religion may buffer psychological effects of mass shootings,^27^ the extant literature on religious coping and spirituality is premised on a Western epistemological paradigm, with measures tending to focus on cognition and behaviour.^26^ By contrast, an Islamic paradigm posits that one's actualising of primordial spiritual purpose through difficulty is central to psychospiritual health, with indigenous conceptualisation of human nature incorporating the spirit/soul.^28^

To our knowledge, no studies have explored the personal experiences of terror attacks on a Muslim population targeted for their shared faith in a non-Muslim majority context. In this study, we aimed to explore effects on health and well-being, connectedness with others and faith, activities of daily living and post-traumatic growth. The Muslim community in Christchurch is diverse, with over 50 countries of origin, inclusive of new migrants and refugee families and those with families in New Zealand for multiple generations.^29^ We hypothesised that the heterogeneity in sociopolitical circumstances and the ethnic and religious intersectionality of this community would contribute to a variety of experiences following the atrocity of 15 March 2019. Previous literature has revealed that secondary stressors and family, work or societal factors unrelated to the incident can compound people's experiences of distress.^13^ We anticipated that the COVID-19 pandemic with its associated lockdowns would be a contributing factor. Furthermore, the ongoing criminal justice process in the context of a highly interconnected faith community may result in more enduring reactions. Another layer to this was the scale of the attacks in relation to the size of the city and small faith community.

## Method

### Research team and reflexivity

Reflecting the importance of Islam for participants, the study team included researchers who were Muslim: a research fellow with experience in international health and trauma research (R.S.-H.); a clinical psychologist (S.D.); and experienced assistant research fellows (F.A. and Z.T.). A clinical psychologist (J.Z.) and an experienced mental health nurse (H.W.) were involved in the design of the semi-structured questions and conducted the qualitative interviews, which allowed for the sensitive content to be discussed in a clinically appropriate manner. The remainder of the research team consisted of two psychiatrists (C.B., a senior researcher with extensive experience in trauma research; and K.E., an early career researcher with qualitative research experience) and a professor of nursing with expertise in qualitative research (M.C.).

### Study design

The COREQ checklist, a guideline for reporting qualitative research, was used in the reporting of this study (Supplementary material 1 available at https://doi.org/10.1192/bjo.2024.791).^30^ The study took place at the Department of Psychological Medicine, University of Otago, New Zealand, and was prospectively registered with the Australian New Zealand Clinical Trials Registry (ACTRN12620000909921).

### Methodological orientation

This qualitative study was designed to enable in-depth exploration of participants’ experiences of the impact of the March 15 mosque attacks, recovery and post-traumatic growth. Given the sensitive nature of the material discussed, we chose to use semi-structured individual interviews. Our process of interpretive thematic analysis followed the method described by Braun and Clarke^31^ and was underpinned by a critical realist theoretical position and an ontological position that assumes that people's words provide access to their particular version of reality.

### Participant selection

Participants were identified via a larger, quantitative study^32^ that aimed to recruit 200 people from the Christchurch Muslim community. Although ethnic diversity was most prominent, with more than 34 nationalities represented, the community was also diverse in terms of age, gender, languages spoken, resident status, educational background, employment status and exposure to previous trauma.^32^ Purposive sampling was used to identify people with varied experiences of 15 March, the impacts and the recovery process.^33^ Participants were given information regarding the qualitative study at the end of this quantitative interview and contacted a week later to ascertain their interest in participating. Members of the research team also suggested participants from the wider Christchurch Muslim community who were subsequently approached. Four people participated only in the qualitative study (and not the quantitative study). The study included only English-speaking participants, and all were members of the Muslim community. On the basis of Braun and Clarke's recommendations for sample size,^31^ our sample consisted of 21 participants.

### Ethical approval

All participants were given written and verbal information regarding the study, were aware of the rationale and provided written informed consent. We used research identification numbers for the digital recordings and transcripts of interviews. All identifying information was removed before storage of the recordings and transcripts in secure online facilities at the University of Otago, Christchurch. Participants received gift vouchers of $30 NZD as a token of appreciation of their time. The authors assert that all procedures contributing to this work comply with the ethical standards of the relevant national and institutional committees on human experimentation and with the Helsinki Declaration of 1975, as revised in 2008. All procedures involving human subjects/patients were approved by The New Zealand Health and Disability Ethics Committee (19/NTA/147).

### Setting

Interviews took place in the research department or in participants’ homes depending on their preference.

### Data collection

The qualitative interviews were conducted in English by an experienced mental health clinician (J.Z. or H.W.) and were between 45 and 60 min in duration. They took place over a 1-year period from September 2020 to August 2021 (18–30 months after the attacks). The semi-structured interviews focused on the two broad aims described above: first, participants’ descriptions of the impact of the March 15 attacks and its effects on day-to-day life; and, second, the experiences of coping, recovery and post-traumatic growth. Interviews began with open-ended questions (e.g. ‘can you please tell me how March 15 has impacted you and your life?’), and prompts were used if needed to develop a richer understanding of participants’ experiences (e.g. social, faith, functioning, health). Interviews were audio recorded for later transcription. No repeat interviews were carried out. Participants were offered the opportunity to review transcripts. Any identifying information was removed from transcripts to ensure anonymity.

### Data analysis

We followed the process of thematic analysis described by Braun and Clarke.^31^ Three authors (S.D., F.A. and either M.C., H.W. or J.Z.) independently read and re-read each interview transcript to become intimately familiar with the data. This was followed by independent iterative generation of initial codes, which were discussed and agreed upon. Codes were then clustered into related ideas to generate themes by four authors (S.D., F.A., Z.T. and M.C.). The relationships of the themes to each other were explored, as were their relationships to the sociocultural context. These themes were discussed among the research team to reach a consensus. According to Braun & Clarke,^34^ the researchers make a situated, interpretative judgement about when to stop coding and move to theme generation and when to stop theme generation and mapping thematic relationships to finalise the written report. A judgement was made in this study that we had identified a cohesive set of themes from the interviews. Quotations supporting the themes and categories are shown. The themes were analysed in relation to each other. Participants often used the terms ‘Allah’ and ‘God’ interchangeably. We have used *shuhada* (singular *shaheed*) for those who died fulfilling a religious commandment in prayer and are thus promised a high position in Paradise.

## Results

The 21 participants included nine men and 12 women. There was a wide range of ages, with participants aged 18–29 (*n* = 5), 30–49 (*n* = 9), 50–69 (*n* = 5) and 70 years and above (*n* = 2). Whereas most participants were either bereaved family, injured, or those who survived without injuries or their family members (collectively referred to as ‘survivors’ in this paper), four support people also participated. Specific numbers for these exposure groups are not reported owing to potential identifiability. Most of the interviews were in person (*n* = 15), with the remaining interviews conducted over Zoom.

Four themes were identified from the qualitative data that related to the participants’ experiences at the time of and following the March 15 attacks: being overwhelmed in the midst of chaos; experiencing silent and enduring impact; living similarly, but differently; and gaining meaning and growth. See Box 1 for themes and subthemes.

**Box 1:** Superordinate themes and subthemes

**1. Being overwhelmed in the midst of chaos** Shock and horror Evolving early response
**2. Experiencing silent and enduring impact** Post trauma reactions Ongoing secondary stressors Barriers to psychological support Family and community relationship dynamics Social and political factors
**3. Living similarly, but differently** Coping strategies and hope Relationships and connectedness
**4. Gaining meaning and growth** Spiritual reality and purpose Social connection Transformation

### Being overwhelmed in the midst of chaos

This major theme was developed from participants’ descriptions of their initial reactions to the attacks. The experiences of shock, horror and fear, as well as strong physiological responses during the attack and in its immediate aftermath, were pronounced. For those who were inside the mosque, there was a sense of the horror of being present and confusion around what was happening.

*I heard a big sound. Actually, I thought that's maybe some(thing else). Then after a few seconds, I understand it was a gunshot. Then I saw him, the gun shooter … And he shot me… I think that people maybe died … I'm not sure we could understand that at the moment (Participant 7)*.

Others watched the devastation unfold on television and social media, which contributed to an initial sense of unreality. Many accidentally came across the live-stream, grappling with disbelief at the nature of the violence and recognising the faces and names of loved ones. Conversely, some participants described an initial feeling of calm and a sense of denial that the attack had happened.

*I was just like, it's not real, this can't be happening, and it just felt like a movie (Participant 18)*.

*[T]hen it was crazy because every single person you saw was someone you knew … then you'd just see the numbers jump from 5 dead, 15, 20 … (Participant 18)*.

For some, it was as if time stood still.

*That day was probably the longest day I've ever had, I remember the day felt like a week … [time] was so slow. It was so intense (Participant 5)*.

The chaos that followed for family and community members when attempting to locate a loved one, was complicated by the scale of the attack, the number of victims, and the extent of police and emergency response.

*It was days before we found out. There was no list. We weren't being kept informed as to who was in hospital, and who had died or anything like that (Participant 3)*

There was an evolving early response in the weeks and months following the attacks. Many participants experienced physical symptoms that affected their daily routines.

*Just lack of motivation, and wanting to sleep, and tiredness … (Participant 9)*

Most described a range of emotional responses, including immense guilt that they were alive when others had died, grief and fear. Over time, participants found that the gravity of what had occurred settled in and the depth of the trauma was acknowledged.

*When I started crying I didn't stop for a couple of days and after that was the funerals, I felt so guilty. It was such a weird feeling to have because why would I be guilty for not being dead if I wasn't even there … (Participant 18)*.

*… we were going through multiple emotions. There was a lot of togetherness, coming together. A lot of healing that was being shown by our community and the global community. But there was deep layers or traumatic experience that was masked underneath that whole sense of coming together and positivity. And it was only after possibly the first few weeks, or even the first month, that it really sat down with people in terms of the gravity of what happened. And for me, it took about a month or so to fully go through the whole process of different areas, different levels of trauma (Participant 11)*.

For most, there was a sense of threat to their safety as being identifiable as Muslim and fear that they could be targeted as an individual or that a further attack could happen.

*Well every time in my mind that thing comes, someone might come. Because the man in the jail now, but you think he hasn't done by himself, so that may continue in my mind all the time. (Participant 12)*.

*… I don't put it on my head while driving to my prayer place where I go for prayer, I don't, if somebody see me, oh he's a Muslim, he's a Muslim. I'm so scared of that, I don't wear that thing … everything is changed now. I can't follow my religious ways (Participant 12)*.

This was exacerbated by social media contributing to the fear and mistrust.

*Comments on social platforms, social networks that were actually not censored at the time of 15 March. That were actually encouraging, and endorsing, and congratulating the shooter … Facebook, Twitter, YouTube … (Participant 11)*

Some had a need to appear strong. For this participant, it was related to their sense of responsibility as the male in the family and a need to be strong for others:

*I'm man, I don't have to show my kind of emotional things to people. I don't want to shed tears, I don't want to cry (Participant 21)*.

After the initial shock and horror, the emotional impact of the killings was varied and associated with strong physical symptoms, including hypervigilance.

### Experiencing silent and enduring impact

This theme captured participants’ descriptions of their attempts to adjust to life alongside the pain of grief and loss. It covers ongoing distress, secondary stressors and the impact on people's lives. A wide variety of experiences were described. There also appeared to be a high level of distress among participants, with the impact altering how everyday life was experienced.

Post-trauma reactions were pervasive. Although there were post-trauma reactions that probably reflected mental illness, there also appeared to be a high level of distress. For some participants, this resulted in avoidance and withdrawal. The ubiquitous impact of the attacks was a strong theme across interviews

*… basically 15 March changed every part of my life (Participant 1)*

The live-streaming of the attack exacerbated the effects of trauma, with many describing flashbacks and other symptoms probably meeting clinical threshold.

*I started getting crazy nightmares about the shooting, crazy, crazy nightmares. I wasn't there that day, and I know that, but maybe it was from watching the videos too many times. I would see his face in the curtains, and then when I'm sleeping, the whole scene would replay in my head (Participant 18)*.

Some participants described masking their distress, including emotional reactivity and low mood, whereas others tried to keep busy as a distraction from their distress. Beneath the togetherness and healing from the global community there is deep pain.

*People tell me … you look great, you look good, and I'm like, well outside, but inside, but inside is a different story (Participant 8)*

*Not once did I allow myself to just sit down and take time to cry. We just pushed ourselves, pushed ourselves the whole time and it was really exhausting (Participant 3)*.

*A lot of healing that was being shown by our community and the global community. But there was deep layers or traumatic experience that was masked underneath that whole sense of coming together and positivity (Participant 11)*

Compassion fatigue was widely experienced, particularly by those in support roles, whether they were family members of the deceased, those who survived the attack or members of the wider community.

*I experienced compassion fatigue … listening to people and their trauma, and their pain and grief … I was just so drained … (Participant 20)*.

Avoidance took different forms for participants and family members and included internal (memories of the day of the attack) and external (the mosque) triggers. One participant said they modified their dress for fear of being identifiable as Muslim. Avoidance appeared to delay help-seeking for some.

*… my husband don't want to turn the TV on, the media and everything (Participant 10)*.

*I was so in denial that I needed any help because I was like I'm okay like I lived. I was still feeling that survivor guilt at the beginning (Participant 1)*.

There were a range of ongoing stressors related to participants’ injuries, financial challenges and disrupted support networks.

*I was finding it hard to go to work with my pain, body pain, and … injury (Participant 12)*.

*I took around like a month off from my work and I was new … I'm not allowed to take days off, so it will be unpaid. So, it put financial pressure on myself (Participant 2)*.

*But knowing that all my support system was going through a rough time, it wasn't appropriate for me to talk. I wish I had got professional help, better professional help (Participant 18)*.

*We've got like literally (few) people who are functioning, everyone else has either been killed (or injured) (Participant 17).*

Participants also described the barriers they encountered in accessing psychological support. Some of this was related to delays in seeking help and the different time-frames involved in accessing the available care.

*There's been lots of times where I could probably go see a counsellor or something, but I think there's not a lot of things in place where you could just quickly pick up the phone and be like I'll make an appointment, or I'll talk to someone … it was just so raw at the time that it was kind of just like the last thing you'd think about is going to see a counsellor … my sister did that and she said that helped her quite a bit, so it was effective for her (Participant 5)*

*Down the line I was falling apart and then that's when I really was like okay maybe I need to go see somebody (Participant 1)*.

Feeling unsafe in accessing the mosque also contributed to limiting access to social support and communal worship.

*I was so unbelievably scared, I started not to go to the mosque … we having our prayer somewhere else … I don't go either of the mosque (Participant 12)*

The complexity of providing and accessing support in an interconnected community was highlighted in many interviews. For some participants, providing support to others took priority above their own distress. Others described support that was not congruent with what they needed.

*Yeah, it was really hard, because you want to cry, yes we cried, I can't hold my tears, but also you have to support someone who really lost someone in his family, which is more important for them more than me (Participant 2)*.

*People were giving me things, at work especially, people were just so generous and they gave me like this massive basket of beautiful things and lots of beautiful messages and then I was like really averse to even being hugged at that point. I like don't offer me your pity, I was really angry about being offered all that love and all that kindness and all that stuff. Personally I was just like I don't want to hug nobody, I don't want to be sad, because I lived (Participant 1)*.

Many participants expressed concern about the impact of enduring distress for family members. These participants disclosed the suffering endured by their husbands owing to being present at the mosque on 15 March and the associated sense of helplessness and personal toll. This affected the participants’ own experiences of distress and social support available to them.

*I experience that after March, he is changed, he was changed. Before we are talking with each other … we are making plans, we want to do this, and this, and everything, but after March he is very quiet, and he is not talking much with me … and (in nightmares) he is shouting and crying in a loud voice (Participant 6)*.

*The hardest thing for me post March is definitely my husband's deteriorating mental health and not wanting to seek help … and then I'm getting worse because he's getting worse, it's inseparable (Participant 1)*.

Another common challenge was community conflict and differences of opinion, which was an ongoing source of distress. This was particularly significant given the small, interconnected nature of the community. In an already diverse community, further categorisation of those affected by proximity to the attacks (e.g. bereaved, injured, witnesses) may have contributed to further fragmentation.

*When it comes to meetings … everyone has a different opinion and it kind of stresses you out (Participant 8)*.

*Whoever was on the list has been defined as a victim, and everybody else hasn't. So more and more you're seeing resources being thrown at that group, and everybody else is oh you weren't there, you don't count … Finances have definitely had a big role in dividing the community, who got what (Participant 3)*

Some participants saw the significance of New Zealand's history of colonisation and the effects of marginalisation and institutional racism in their own experiences of racism and discrimination before and following the attacks.

*It was really terrifying to read comments (social media), and they were like oh should have killed them all, wish there was more, I'm celebrating. But you don't necessarily see all these people in the street, because their hatred is hidden (Participant 18)*.

*There will always be Government agencies whose job it is to support those who are affected, but that may be a step removed from a sense of trust (Participant 11)*.

*The Treaty of Waitangi and the effects of marginalisation … I've experienced what it must have been like to be Maori on a very small scale. And people say things like this is the worst massacre in modern day history, it's never happened like this, and I'm thinking, yes it has. Colonisation has created annihilation of community and culture … we still have a lot of system institutionalised racism and discrimination, stereotyping (Participant 9)*

Despite this, there were reflections on a greater connectedness and awareness in the wider Christchurch community. Improved awareness and understanding of Islam and Muslims led to participants feeling that their identity and beliefs were more accepted and understood, and some people became more comfortable with being visibly Muslim as a result.

*I'm proud of the way that New Zealanders responded. I thought that was just amazing … it's kind of nice now that people know more about Islam, and more about Muslims (Participant 3)*.

*But then after I'd find myself telling my friends, I'm just going to go pray, and it was so much easier for them to understand, and it was good after that. There's more awareness, people know about Muslims, it's been good (Participant 18)*.

There were strong perspectives on the media expressed by participants. Some criticised reporting that reinforced stereotypes about Muslims or was intrusive, or that depicted survivors’ experiences as homogenous. Participants described a need for a more individualised understanding of experiences. However, efforts made by the New Zealand media to report in a sensitive manner following 15 March were also commended.

*Everywhere you went there were cameras in your face (Participant 3)*.

*I think the New Zealand media has been so good when it comes to reporting about the terrorist and things like that, but when it came to reporting about opinions, then there is room for improvement there. So, the media would speak to one or two … would say that they are representatives of* the Muslim community, *but it doesn't reflect on everyone's opinions. And that really got to me (Participant 8)*.

*Stuff (New Zealand media organisation) … have since then had an overhaul of their privacy and … ethical approaches towards journalism. Which has kind of reassured me … they are doing their part in trying to combat hate speech … that could have been done much earlier, it didn't need to take 15 March to recognise that hate speech (Participant 11)*

The unprecedented criminal justice sentencing in New Zealand that occurred in August 2021 and the court processes leading up to it had an impact on participants’ well-being. The announcement of a guilty plea while the nation went into the first COVID-19 pandemic lockdown, attending court, and giving and viewing victim impact statements during the sentencing hearing was overwhelming or came with frustration and relief for some. There was a diversity of impact, with some participants describing an exacerbation of distress and increased isolation in this period, whereas some noted the significance of the terrorism charge in terms of feeling validated, and others experienced the facilitation of access to the legal process as culturally responsive. Victim impact statements and court attendance come with an opportunity to be heard, combined with the risk of retraumatisation.

*I think for me it was a bit overwhelming because it was never dealt with the court in my life, first I'm going to court getting involved with police and the statement helping them out with all that and it was very overwhelming (Participant 4)*.

*I was present during those four days (of sentencing), and there was a lot of memories that had been revisited, and I had to revisit as well as a lot of stories I saw, I heard. And that made me upset, angry (Participant 11)*.

*They got better, because they were good about asking for feedback and so, I smiled when they did sentencing, which they actually did a great job of eventually, where they suddenly this very Westernised building, had a version of cultural acceptance, because they'd provided a prayer space and wellbeing area for us to go to do our prayers during the day (Participant 9)*.

Some saw no benefit in engaging with justice processes:

*I was not interested in that part. It just prolonged the agony and misery; it didn't help anything (Participant 14).*

One participant noted the weight of being Muslim as synonymous with being a terrorist in the eyes of society, something that contributed to the value placed on the terrorism charge.

*(Prior to 15 March) I didn't want to be judged or recognised as a Muslim and what they might think, will they think I'm a terrorist? … (Participant 9)*.

*The Prime Minister said it was like a terror attack, and that want Muslim people want to hear, terrorist. Because we always like, Muslim, you know the media, terrorist, and we are not (Participant 21)*.

The COVID-19 pandemic and lockdown that was associated with it occurred as the perpetrator pleaded guilty and was sentenced. There was overwhelm and isolation experienced with this.

*… there were times being at home, there were times when I just wanted to take a break, you know when you're facing each other 24/7, the tensions over small things, ridiculously small things, make you lose the plot really (Participant 8)*.

*He pleaded guilty at about 10 or 10.30 or something, and the media released it in less than an hour … So, we weren't told properly, and we weren't anticipating it. And we're all in a lockdown, away from everyone. I couldn't even go to see my family to be with them when we heard to even debrief and talk about it (Participant 9)*.

For some, there was an overwhelming sense of relief once the sentencing was over.

*When it got to the sentencing, after that, that was like a cloud lifting (Participant 19)*.

### Living similarly, but differently

This major theme refers to the ways in which life remained outwardly the same for many participants; however, alongside this was a sense that things had changed forever. Many described how their faith brought comfort and the ability to continue with life. There was a sense that life was generally back to a ‘new-normal’ rhythm, viewing life with fresh perspective, actively seeking psychological support and consciously engaging in faith practices. A sense of hope underpinned this.

Participants identified the importance of self-care in the recovery process. Some expressed becoming intentional about lifestyle and faith practices, whereas others described how they looked after themselves in order to improve their well-being. Some participants sought professional input as part of self-care and recovery.

*I've realised I've got to take care of myself in order to give to others (Participant 20)*.

*Re-prioritising, re-thinking what I want to do, and what my life will involve … I've reduced a lot of my workload … And just kind of chosen to do what I love (Participant 9)*.

*I've committed going there (gym) every class, rain or shine, no matter how tired I am. Like I say, I surprised myself, I don't even know how I did it (Participant 8)*.

*I see a psychologist and I just do whatever she suggests and it's helping me function, basically (Participant 1)*.

*… we would go to any Mosque … pray, you have some people to talk to, recite Quran, you spend like a few minutes or 15, 20. Peaceful, very peaceful (Participant 21)*.

Some expressed a renewed appreciation for life:

*That I noted that there's a difference in me and I'm cherishing every … I read a book, I play some things like games, go for a walk … I start actually running this morning (Participant 4)*.

Although the strain and breakdowns of relationships were discussed earlier, there was a clear theme around healing and strength that occurred through an interconnectedness of personal relationships with God, family and community.

*A lot more closeness with my Dad, my sister, and family overseas that were not together. Definitely a sense of closeness, but also within the Muslim community (Participant 20)*.

*Definitely spiritual … this was meant to happen for whatever reason. It gives me a lot of solace, and peace … I will speak more, much more than I used to. I raise my voice a bit more about things that I don't think are okay. And some of them are discriminatory, or unfair, or inequitable (Participant 9)*.

*I do have a good footing in both my identity, my connection to my faith, as well as my connection to Christchurch. So, I do very much have just as much affection and love for Christchurch. Possibly even more so than before because of the resilience that Christchurch has gone through, not just on 15 March, but all of the other traumatic events leading up to that (Participant 11)*.

The way forward for this participant is coming together and not letting the terrorist act define them.

*It's how do we collectively, cooperatively, productively, positively, turn this into something where we say you cannot let the crap define you (Participant 19)*.

### Gaining meaning and growth

This theme describes how the participants were able to live in a meaningful way despite the pain and loss and were able to strengthen connections with family, community and faith. It captured a rich tapestry of self-discovery, life perspective and spiritual engagement.

The notion in the Islamic tradition of the trajectory of the soul and existence of a spiritual domain that endures beyond the material world^28^ served as a source of acceptance and perspective for participants.

*And post-15 March it made me realise that a lot of the statement of the noble prophet (Muhammad P.B.U.H.), who we take as an example, a role model in life, not just in terms of religious and ritual acts, but also understandings based on why certain events happen and why certain calamities happen. And so, there is where I believe myself and many other Muslims who do follow the principles of prophet Muhammed, may peace be upon him, will draw some of those principles of patience and understanding (Participant 11)*.

Referencing the Quran, the prophetic tradition and one's awareness of God as a source meaning was common.

*One of my friends messaged me a verse from Qur'an which basically reflected on the situation … do not think those who are killed in the way of God are dead, but they are alive and well-fed in heaven. So that just gave me content; that he is in good place right now … everything happens for a reason and he was chosen to go to heaven (Participant 8)*.

*And that's something I'm really thankful for. So many opportunities for growth and learning (Participant 18)*.

Many were able to participate in collective meaning-making and growth through their social life. For some, the support of community religious leaders including Imams was important.

*Imam said: we are not going to retaliate, our heart is going to outweigh the kindness that we have … justice will prevail in its own way. We shouldn't do anything (Participant 16)*.

Being able to go on a journey of recovery with family, friends and community was important.

*For me, number one would be family, having a strong family here who are very supportive and who are going through the same journey. And being able to support them, and them supporting me, having that two-way (Participant 11)*.

*I was exposed to a lot more of the community that I may have seen at the mosque or at the Muslim community events but had never engaged with. Suddenly we were all so close (Participant 20)*.

A sense of social responsibility provided purpose. The Islamic tradition was an impetus for serving others.

*We weren't there for a reason, and because we weren't there that day, we have a responsibility to step up and do what you can. And there's very much this thought in Islam … if you're in a situation where you can do something, you should do it (Participant 3)*.

*We see teachings in Islam that are profoundly beautiful … they're life-affirming. They say you have a responsibility as a created being towards everything else in the world (Participant 19)*.

Whereas clear references to cognitive, behavioural and social aspects of religious coping were noted above, for some, there appeared to be a transformative, experiential process through their faith, from ‘going through the motions’ to engaging fully with the pain through reflection (turning inwards) and more intentional faith practice.

*… at the start, I did lose my faith. Growing up, you're just taught your religion, you're just taught your faith, but you don't really question … And so, that's been such an awesome journey for me to rediscover my faith, and I'm so much happier (Participant 18)*.

*So, my parents pray, so I pray, and fasting, I'm fasting, so we grow up like that. But after the incident, I learn more about it. Search more and it give me and I think most of the community, more stronger … it's getting much, much, much, much stronger than before (Participant 2)*.

## Discussion

This qualitative study of interviews with 21 participants 18–30 months after the terrorist attacks on two mosques in Christchurch, New Zealand, produced rich insights into the experience of a large-scale attack on a diverse minority faith community. The four themes identified in the data interacted with each other in a non-linear manner. After the initial shock and horror, the impact of the killings was varied. Strong emotional experiences including guilt and fear were common and often associated with physical symptoms including hypervigilance and disrupted sleep. Generally, participants found a new rhythm with living despite enduring post-trauma reactions and ongoing secondary stressors. Difficult family and community dynamics and social withdrawal occurred alongside the strengthening of family and community connections for many participants. Individual and relational coping was associated with recovery, with meaning-making and post-traumatic growth characterised by enhanced self-awareness and spiritual engagement. These findings have commonalities with existing literature but also unique aspects related to the context of the trauma. Distinctive aspects of our findings relate to the strong spiritual dimension in meaning-making and personal growth. The compounding effects of the context of a targeted attack on a diverse minority faith community and particular secondary stressors, such as the COVID-19 pandemic and justice processes, were also unique. Consistent with previous findings, there was acute distress in the immediate aftermath of the attacks and prolonged grief and pervasive post-trauma reactions, often exacerbated by secondary stressors. Also similar to previous findings was the importance of individual, relational and spiritual elements in recovery and post-traumatic growth.

### Centrality of faith/Islam

The depth of the interconnection between mental health recovery and post-traumatic growth was a defining feature of our findings. There was a strong spiritual thread in personal narratives following the attacks that wove together these experiences of recovery and growth.

The vast majority of participants described a strong awareness of God and metaphysical reality regardless of level of distress. The notion of *Qadr* (predestination) provided a sense of comfort and acceptance, where participants were able to put their experiences in the context of a wider plan. Religious coping was an element of the spiritual engagement described by almost all participants. Although there were clear references to cognitive, behavioural and social aspects of religious coping, as noted above, for some there appeared to be a transformative, experiential process that required grappling with painful emotional states and simultaneous connection to God. This highlights the need for therapeutic approaches to include a focus on enhancing positive religious coping and maximising the potential for spiritual growth as a resource.^28^

### Compounding factors

Some features of the context in which the attacks occurred that were not stressors in themselves or were unrelated to the events compounded the effects of the primary and secondary stressors. The diverse make-up of the Muslim community that was targeted by the perpetrator, together with the social and political contexts, the overlapping COVID-19 pandemic and the justice processes appeared to have important implications for survivors’ recovery and post-traumatic growth. Previous studies^7^ have also shown that survivors from ethnic minorities are more vulnerable, although reasons for this are not fully understood.

### Diversity, size and interconnectedness of community

As we noted above, during times of distress, people often turn to family and friends for support.^14^ However, when the affected community is small and interconnected, a sense of responsibility may be held by a relatively small number of people providing support. This results in inadequate social support owing to the debilitating impact of the attack on what would typically be considered ‘natural support’ (i.e. close family and community members). The theme of people commonly turning to families and friends for support and particularly valuing connections with those with a shared understanding of their experiences has been consistently reported.^14^ Previous studies^13^ have found that people minimise their needs compared with the perceived needs of others. In our study, this appeared to be the case regardless of proximity to the attacks or deceased and was reflected by reluctance to seek both personal and professional support. Understanding this support and the potential consequences for those providing it has received little attention to date. It has also been shown that although social networks have a stress-buffering effect, when this support is perceived as lacking, avoidant or inept,^14^ there can be negative or stressful impacts. Our findings emphasise the need to further examine the complexity of social support in the context of highly exposed small and interconnected communities.

Post-trauma impacts, coping and recovery, even within such a small subsection of those affected by the atrocity of 15 March, were characterised by complexity and diversity. Participants described exacerbation caused by having individual circumstances and stories generalised to those of the ‘survivor group’, having been on the receiving end of this from community members, media, health professionals and the general public. The desire to be ‘seen’ as a unique and nuanced individual was important, and this is a phenomenon expressed by members of ethnic minority groups in healthcare settings.^20^

Similarly, the experience of professional mental health support was varied. A number of participants described therapeutic benefits from engaging in psychological intervention, whereas others continued to experience barriers to access including ease, timing and goodness of fit with the therapist. One participant noted that they would only see a therapist of Muslim faith, whereas another stated that they explicitly did not want this. Yet others did not attempt to seek professional support. Help-seeking may have been further compounded in this context by concerns about confidentiality and mental health stigma. Overall, findings reinforced that post-trauma impact and need are multifaceted and varied despite the interconnectedness of this minority community.

### Social and political context

Some participants in our sample reported feeling very identifiable following 15 March and therefore at risk of discrimination or further attacks. Many also felt that there was an increased understanding of Islam within the wider New Zealand community. This may have reflected the general public's initial response to the attacks, which involved an outpouring of support and warmth toward the community.^35^ These contrasting experiences tended not to be mutually exclusive.

Some participants reflected on the social and political context of the attacks, which specifically targeted a minority population. There were references to institutional racism and impact of colonisation in general, as well as prior personal experiences of cultural insensitivity and discrimination by government agencies. This social and political context within which the attacks happened seems to have had an impact on people's experiences of post-attack responses and their effectiveness. Where negative stereotypes of the community that participants represented were challenged, it led to positive effects on experiences of distress. This is illustrated by the significance of early labelling of the attack as a terrorist attack by authorities and the terrorism charge that was added to charges facing the offender, providing relief from the burden of carrying the ‘terrorist’ label, especially for Muslim men, who felt that the title was finally being correctly assigned.

### Distress in the acute phase and longer-term post-traumatic impact

Consistent with existing literature, we report wide variability in the symptoms of distress experienced in the aftermath of a traumatic event. These ranged from fear, shock and horror to a sense of calm and, for some, gratitude, emphasising the importance of understanding that acute distress can be experienced in many different ways.^13^ Previous qualitative studies have also noted expressions of distress including fear,^12,15,17^ although some have not found the same degree of shock that participants in our study experienced.^13^ It is possible that this relates to the unprecedented nature of such an attack in New Zealand, and the scale of the attack and associated confusion, cordoning of the mosques, and delays in identifying the deceased probably contributed to the severity of post-trauma responses.

Consistent with the literature, some participants in our sample also reported feelings of guilt,^12,13^ which has been shown to affect the intensity and trajectory of distress after trauma.^13^ This suggests that broadening assessments to include these emotions and potentially targeting them in interventions may be important. As previously demonstrated, somatic symptoms were widely experienced, ranging from hypervigilance to disrupted sleep.^7,13^

Similar to findings following the 2017 Manchester Arena bombings, enduring distress was prevalent and often inconsistent with participants’ and society's expectations of the trajectory of grief.^13^ Our data revealed the experience of prolonged grief and, in some cases, functional impairment for both the interviewee and their family. This was pronounced for those who had lost a loved one or were survivors of the attacks, or lived with a family member who was present at the mosque on 15 March. As has been previously reported,^14,16,22^ for some, there was a sense of being under continued threat and danger, related to assumptions of safety being shattered.^36^

### Role of secondary stressors

Our findings broadly support existing literature demonstrating the powerful impacts of secondary stressors on recovery. For participants, these included strain on close relationships, financial pressure or protracted legal processes. Notably, inappropriate or invalidating forms of social support may serve as secondary stressors. Although specific individual stressors were discussed, there were also shared stressors such as media reporting and justice processes. A number of participants reported concerns in multiple areas of their lives, and many of these were complex and exacerbated by compounding factors.

The time-frame for data collection captured the significant secondary stress experienced by participants. The literature on the impact of criminal justice processes on disaster response and recovery from terrorist attacks is limited. However, our findings suggest that this exacerbated post-trauma reactions. Going into a COVID-19 lockdown less than a year after the attacks, while many participants were still struggling with immigration issues and reuniting with family from overseas, compounded the limited social support available. Meanwhile, an unexpected guilty plea during the COVID-19 pandemic lockdown added to distress. Participants expressed frustration and distress related to media reporting that reinforced stereotypes or reflected a homogeneous view of the survivors’ experience. This was mitigated to some degree by sensitive and nuanced media reporting. Similarly, many participants found court processes retraumatising and difficult to navigate. However, this appeared to be softened by culturally responsive measures in court and the opportunity to engage in sharing perspectives via victim impact statements. Whereas some were uninterested and disengaged from the justice process, others described immense relief following the sentencing.

### Post-traumatic growth

Participants in our study described trying to adjust to life alongside the pain of grief and loss. This theme of finding meaning in the pain has been reported in other studies.^15^ In line with emerging post-traumatic growth literature, the experiences of distress and meaning-making were not mutually exclusive.^37^ Participants referred to growth involving enhanced self-awareness and self-care, fostering of relationships and a meaningful life perspective. Of significance was the emphasis placed on spiritual engagement. This is consistent with previous studies which report that following exposure to traumatic events, some people cope by ‘turning to religion’ or experience a strengthening of a personal relationship with God.^38^ In particular, previous findings highlighting the importance of framing post-disaster experiences in religious terms^23^ was observed in our sample. This included concepts of the centrality of God and a metaphysical reality where the soul endures beyond the temporal world,^28^ reflected in notions of the status of a martyr (*Shaheed*), predestination (*qadr*) or the belief that God is merciful and has a bigger plan. Similar themes relating to a sense of meaning despite grief have been reported in qualitative studies involving Muslim survivors after natural and man-made disasters^22,36^ and among Muslim refugees.^39^ An Islamic perspective was reflected on by varying degrees by participants, with most describing faith as providing solace, meaning and perspective. For a smaller number of participants, this was not the case. Some participants reported making sense of their experiences through a shift in priorities where they placed value on different things in life, particularly relationships, work and community. This was consistent with the findings of other studies.^16^

The importance of social connection in relation to resilience and positive outcomes has been consistently reported in both quantitative and qualitative studies.^3,12–14,16^ Having good social networks is a key predictor of recovery after major incidents.^40^ Turning to families and friends for support, as well as valuing connection with those with a shared understanding of their experiences, has been previously reported.^14^ Conversely, social withdrawal has been linked to negative changes in functioning.^13^

These findings emphasise the importance of facilitating social connection after traumatic events and of identifying people with few social supports or social withdrawal.

### Strengths and limitations

This study had a number of strengths. It is, to our knowledge, the first study to explore the experiences of a diverse minority Muslim community following a terrorist attack. It is one of the few studies to examine subjective experiences of a large-scale mass shooting. It also included members of the wider Christchurch Muslim community to understand effects on people exposed to a spectrum of impact. Interviews were conducted by experienced mental health clinicians, allowing for the sensitive content discussed in interviews to be elicited in a clinically appropriate manner. Our research group came from a variety of backgrounds and included Muslim researchers and clinicians, ensuring broad expertise throughout the study process.

A limitation relating to generalisability was that most participants were proficient in English and interested in speaking further about their experiences. Although many were directly affected or from families of the deceased or injured, almost half were in support roles and part of the wider Muslim community. The small sample did not allow exploration of the nuanced differential experiences of these groups. Therefore, the themes may not fully reflect the full breadth of experiences. For example, those who were more isolated may have been less likely to take part in the quantitative study from which the sample was drawn. The relatively wide time-frame within which the interviews were conducted and the associated impacts of the COVID-19 pandemic and the criminal justice process may also have influenced findings. Prompts in the interview schedule were directed at exploring a number of aspects that were thought to have been potentially affected by March 15 and may not have captured all relevant issues. Finally, Islam is viewed as a living tradition integrating spiritual, cognitive and social dimensions. As such, all forms of coping would be viewed as incorporating a spiritual element; therefore, using concepts such as ‘religious coping’ may not be appropriate in this and other contexts where religion and culture are more closely intertwined.

### Implications and recommendations

This study adds to a growing body of literature pointing to ongoing post-trauma reactions and post-traumatic growth associated with terror attacks. The susceptibility to adverse, widespread and individualised mental health outcomes appears to be universal, as are the meaning-making, fostering of relationships and spiritual engagement that can occur as a result. However, the centrality of faith to the relationship between mental health recovery and post-traumatic growth is a novel finding. This highlights the relevance of developing conceptualisations of trauma response, religious coping and recovery grounded in indigenous, in this case Islamic, paradigms.^26,28^ In addition, the diversity of this interconnected community, together with compounding impacts of COVID-19 and the social/political context, reflects an added layer of complexity and need. Our results also provide unique insights into the secondary stressors and nuanced recovery and needs of a small and multicultural minority faith population.

The heterogeneity of participant experiences in this study points to the need for greater choice and resources to support personalised, relational and spiritual aspects of recovery. Psychosocial responses need to reflect the multifaceted experiences of those at the centre of the atrocity. This includes specialist mental health services with community level support and consideration of the needs of the individual and family. Our findings are a reminder that well-established principles of trauma-informed care, such as choice, empowerment and caring interactions, are paramount. Facilitating access to health and justice processes may also mitigate barriers to recovery. We recommend that future studies use qualitative alongside quantitative approaches to better understand the longer-term support needs and recovery trajectory of those affected by terrorist attacks.

## Supporting information

Dean et al. supplementary material Dean et al. supplementary material

## Data Availability

The data from the qualitative interviews are not publicly available because they contain material that could potentially identify participants.

## References

[1] Wilson N, Thomson G. Mass shooting in Christchurch and the epidemiology of sudden mass fatality events in New Zealand. N Z Med J 2019; 132(1494): 68–70. 31048828

[2] Santiago PN, Ursano RJ, Gray CL, Pynoos RS, Spiegel D, Lewis-Fernandez R, et al. A systematic review of PTSD prevalence and trajectories in DSM-5 defined trauma exposed populations: intentional and non-intentional traumatic events. PLoS One 2013; 8(4): e59236. 23593134 10.1371/journal.pone.0059236 PMC3623968

[3] Rigutto C, Sapara AO, Agyapong VIO. Anxiety, depression and posttraumatic stress disorder after terrorist attacks: a general review of the literature. Behav Sci (Basel) 2021; 11(10). 10.3390/bs11100140 PMC8533613 34677233

[4] Kristensen P, Dyregrov K, Gjestad R. Different trajectories of prolonged grief in bereaved family members after terror. Front Psychiatry 2020; 11: 545368. 33192660 10.3389/fpsyt.2020.545368 PMC7591785

[5] Dyregrov A, Salloum A, Kristensen P, Dyregrov K. Grief and traumatic grief in children in the context of mass trauma. Curr Psychiatry Rep 2015; 17(6): 48. 25940038 10.1007/s11920-015-0577-x

[6] Bharadwaj P, Bhuller M, Løken KV, Wentzel M. Surviving a Mass Shooting. National Bureau of Economic Research, 2021.

[7] Dyb G, Jensen TK, Nygaard E, Ekeberg O, Diseth TH, Wentzel-Larsen T, et al. Post-traumatic stress reactions in survivors of the 2011 massacre on Utoya Island, Norway. Br J Psychiatry 2014; 204: 361–7. 24115344 10.1192/bjp.bp.113.133157

[8] Chandrashekar S. Engendering threat in the guise of protection: orientalism and Sikh vulnerability. J Multicult Discourses 2017; 12(4): 366–81.

[9] Mahrouse G. Minimizing and denying racial violence: insights from the Québec mosque shooting. Can J Women Law 2018; 30(3): 471–93.

[10] Weber E, Miller SJ, Astha V, Janevic T, Benn E. Characteristics of telehealth users in NYC for COVID-related care during the coronavirus pandemic. J Am Med Inform Assoc 2020; 27(12): 1949–54. 32866249 10.1093/jamia/ocaa216 PMC7499577

[11] Imtiyaz ARM. The Easter Sunday bombings and the crisis facing Sri Lanka's Muslims. J Asian Afr Stud 2019; 55(1): 3–16.

[12] Wilson N, d'Ardenne P, Scott C, Fine H, Priebe S. Survivors of the London bombings with PTSD: a qualitative study of their accounts during CBT treatment. Traumatology 2012; 18(2): 75–84.

[13] Stancombe J, Williams R, Drury J, Collins H, Lagan L, Barrett A, et al. People's experiences of distress and psychosocial care following a terrorist attack: interviews with survivors of the Manchester arena bombing in 2017. BJPsych Open 2022; 8(2): e41. 35109959 10.1192/bjo.2022.2 PMC8867861

[14] Dyregrov K, Kristensen P, Dyregrov A. A relational perspective on social support between bereaved and their networks after terror: a qualitative study. Glob Qual Nurs Res 2018; 5: 2333393618792076. 30116765 10.1177/2333393618792076 PMC6088469

[15] McCormack L, McKellar L. Adaptive growth following terrorism: vigilance and anger as facilitators of posttraumatic growth in the aftermath of the Bali bombings. Traumatology 2015; 21.

[16] Bauwens J. Losing a family member in an act of terror: a review from the qualitative grey literature on the long-term affects of September 11, 2001. Clin Soc Work J 2017; 45(2): 146–58.

[17] Hawkins NA, McIntosh DN, Silver RC, Holman EA. Early responses to school violence. J Emot Abuse 2007; 4(3-4): 197–223.

[18] Mennecier D, Hendrick S, Mol J, Denis J. Experience of victims of Brussels’ terrorists attacks: an interpretative phenomenological analysis. Traumatology 2020; 30(1): 86–96.

[19] Sheikhi R, Seyedin H, Qanizadeh G, Jahangiri K. Role of religious institutions in disaster risk management: a systematic review. Disaster Med Public Health Prep 2020; 15: 1–16. 10.1017/dmp.2019.145 32063259

[20] Ridge D, Pilkington K, Donovan S, Moschopoulou E, Gopal D, Bhui K, et al. A meta-ethnography investigating relational influences on mental health and cancer-related health care interventions for racially minoritised people in the UK. PLoS One 2023; 18(5): e0284878. 37163472 10.1371/journal.pone.0284878 PMC10171693

[21] Platt JM, Lowe SR, Galea S, Norris FH, Koenen KC. A longitudinal study of the bidirectional relationship between social support and posttraumatic stress following a natural disaster. John Wiley Sons 2016: 205–13. 10.1002/jts.22092 PMC5800398 27163339

[22] Lodhi S, Gul S, Khattak A. A qualitative study on posttraumatic growth processes in trauma victims: evidence from Pakistan. Psychiatr Danub 2022; 34: 263–72. 35772136 10.24869/psyd.2022.263

[23] Davis EB, Kimball CN, Aten JD, Andrews B, Van Tongeren DR, Hook JN, et al. Religious meaning making and attachment in a disaster context: a longitudinal qualitative study of flood survivors. J Posit Psychol 2019; 14(5): 659–71.

[24] Kucharska J. Religiosity and the psychological outcomes of trauma: a systematic review of quantitative studies. J Clin Psychol 2020; 76(1): 40–58. 31557330 10.1002/jclp.22867

[25] Aten JD, Smith WR, Davis EB, Van Tongeren DR, Hook JN, Davis DE, et al. The psychological study of religion and spirituality in a disaster context: a systematic review. Psychol Trauma 2019; 11(6): 597–613. 30730187 10.1037/tra0000431

[26] Voytenko VL, Pargament KI, Cowden RG, Lemke AW, Kurniati NMT, Bechara AO, et al. Religious coping with interpersonal hurts: psychosocial correlates of the brief RCOPE in four non-western countries. Psychol Relig Spirituality 2023; 15(1): 43–55.

[27] Roman L, Mosher D, Hook J, Captari L, Aten J, Davis E, et al. Religious support buffers the indirect negative psychological effects of mass shooting in church affiliated individuals. Psychol Trauma 2019; 11. 10.1037/tra0000448 30843716

[28] Rothman A. Developing A Model of Islamic Psychology and Psychotherapy: Islamic Theology and Contemporary Understandings of Psychology. Routledge/Taylor & Francis Group, 2022.

[29] Drury A. Wish you were here; a short history of New Zealand Muslims and integration. Nazhruna 2020; 3: 355–70.

[30] Tong A, Sainsbury P, Craig J. Consolidated criteria for reporting qualitative research (COREQ): a 32-item checklist for interviews and focus groups. Int J Qual Health Care 2007; 19(6): 349–57. 17872937 10.1093/intqhc/mzm042

[31] Clarke V, Braun V. Successful Qualitative Research: A Practical Guide for Beginners. SAGE, 2013.

[32] Sulaiman-Hill R, Porter R, Tanveer S, Boden J, Beaglehole B, Schluter P, et al. Psychosocial impacts on the Christchurch Muslim community following the March 15 terrorist attacks: a mixed-methods study protocol. BMJ Open 2021; 11(10): e055413. 10.1136/bmjopen-2021-055413 PMC8488282 34598996

[33] Palinkas L, Horwitz S, Green C, Wisdom J, Duan N, Hoagwood K. Purposeful sampling for qualitative data collection and analysis in mixed method implementation research. Adm Policy Ment Health 2015; 42: 533–44. 24193818 10.1007/s10488-013-0528-y PMC4012002

[34] Braun V, Clarke V. To saturate or not to saturate? Questioning data saturation as a useful concept for thematic analysis and sample-size rationales. Qualitative research in sport. Exerc Health 2019; 13: 201–16.

[35] Byrne KG, Yogeeswaran K, Dorahy MJ, Gale J, Afzali MU, Bulbulia J, et al. Psychological impact of far-right terrorism against Muslim minorities on national distress, community, and wellbeing. Sci Rep 2022; 12(1): 1620. 35102221 10.1038/s41598-022-05678-x PMC8803852

[36] Freh FM, Dallos R, Chung MC. An exploration of PTSD and coping strategies: response to the experience of being in a bomb attack in Iraq. Traumatology 2013; 19(2): 87–94.

[37] Mike S, Stefan R-E, Laura B, Joy L-B, Donna F, Ada H, et al. Post-traumatic growth in mental health recovery: qualitative study of narratives. BMJ Open 2019; 9(6): e029342. 10.1136/bmjopen-2019-029342 PMC6609070 31256037

[38] Lee MH, Raitt J, Hong BA, Diduck A, Nguyen AMTT, Villareal A, et al. Making meaning of disaster experience in highly trauma-exposed survivors of the Oklahoma city bombing. Traumatology 2022; 28(2): 202–10. 36035619 10.1037/trm0000326 PMC9400919

[39] Şimşir Z, Dilmaç B, Özteke Kozan Hİ. Posttraumatic growth experiences of Syrian refugees after war. J Humanist Psychol 2018; 61(1): 55–72.

[40] Kaniasty K. Social support, interpersonal, and community dynamics following disasters caused by natural hazards. Curr Opin Psychol 2020; 32: 105–9. 31445427 10.1016/j.copsyc.2019.07.026

